# Callous-Unemotional Traits and Social Adjustment among Chinese Preschoolers: The Moderating Role of Teacher-Child Relationship

**DOI:** 10.3390/ijerph20043426

**Published:** 2023-02-15

**Authors:** Jingjing Zhu, Xiaoying Xia, Qianqian Wu, Shiyao Zou, Yan Li

**Affiliations:** 1 Shanghai Institute of Early Childhood Education, Shanghai Normal University, Shanghai 200234, China; 2 School of Early Childhood Education, Shanghai Normal University Tianhua College, Shanghai 201815, China; 3 Wuchang Experimental Kindergarten, Wuhan 430061, China

**Keywords:** preschoolers, callous-unemotional traits, social adjustment, teacher-child relationship

## Abstract

Callous-unemotional (CU) traits are associated with social adjustment difficulties, but few studies have examined the underlying mechanisms in Chinese preschoolers. This study examined the relationship between CU traits and social adjustment among Chinese preschoolers as well as the moderating role of the teacher-child relationship in the association. Participants were 484 preschool children aged 3–6 years old from Shanghai, China (*M*age = 5.56 years, *SD* = 0.96 years). Parents reported children’s CU traits and teachers reported their relationship with children and rated children’s social adjustment as well. The results revealed that (1) children with higher CU traits positively related to aggressive and asocial behavior with peers, but negatively related to prosocial behavior; (2) the teacher-child relationship moderated the relationship between CU traits and social adjustment in children. Specifically, teacher-child conflict exacerbated the aggressive and asocial behavior of children with CU traits and reduced the prosocial behavior of children with CU traits. These findings extended the current research on CU traits and had important implications for early interventions targeted at children with CU traits.

## 1. Introduction

Callous-unemotional (CU) traits refer to the personality disorders that individuals display in emotions and interpersonal communications. Individuals with CU traits display low levels of guilt and regret, lack empathy, and show indifference to others’ feelings and behaviors [1,2]. Research showed that children with CU traits have difficulty in recognizing painful emotions in others and tend to exhibit mean behaviors and fearless temperament traits [3,4,5]. A body of research has shown that CU traits are associated with severe and stable problem behaviors throughout children’s development. For example, meta-analyses have shown that CU traits are significantly and positively associated with conduct problems for children under 5 years of age [6]. Similarly, prospective longitudinal studies confirm that children with high and stable CU traits tend to exhibit the worst behavioral outcomes [7,8]. Therefore, CU traits are an important risk factor for children’s development [9]. However, previous research has mostly sampled school-age children and adolescents [10]. This is unfortunate because CU traits are already present in preschool [11]. Moreover, conduct problems such as aggressive and antisocial behaviors in early childhood also originate in the preschool period [12,13], and this may be the developmental period in which preventive interventions are particularly successful [14]. Thus, it is critically important to explore CU traits in young children and provide empirical evidence for early interventions targeted at improving the social adjustment of children with CU traits.

### 1.1. CU Traits and Children’s Social Adjustment

As a personality trait, CU traits are potential risk factors for children’s social adjustment problems. Social adjustment is understood as individuals’ adjusted behaviors toward the changing circumstance in their environment to achieve harmony between the individual and environment [15]. In the present study, we focused on four social adjustment behaviors (e.g., aggressive behavior, prosocial behavior, asocial behavior, and peer exclusion) which reflect children’s performance in social interaction. Aggressive behavior is a type of behavior that may harm peers, including physical and verbal aggression [16]. Prosocial behavior refers to the tendency for children to empathize and cooperate with peers [16]. Asocial behavior involves the tendency of children to pursue solitude rather than social interaction in a peer environment [16]. Peer exclusion refers to neglect, rejection, and exclusion by peers [16]. These four constructs are widely used as indicators of social adjustments [17,18,19]. Research indicated that CU traits were associated with numerous social adjustment difficulties in children [10], including disruptive behavior (e.g., aggressive behavior and disciplinary problems), low social competence, and peer problems [7,20,21]. For example, Kimonis et al. found that CU traits were significantly and positively associated with aggressive behavior in preschool children [22]. A meta-analysis by Waller et al. showed that CU traits were negatively related to children’s prosociality [23]. Longitudinal research also reported similar results. For example, a longitudinal study by Waller et al. indicated that children’s CU traits at age three predicted their high levels of aggression and peer exclusion at age ten [24]. Children with CU traits already exhibit emotional recognition difficulties, moral-emotional deficits, and fearlessness in childhood [23,25,26]. However, these features are often associated with less prosocial behavior and more externalizing behavioral problems in young children [27,28,29]. In recent years, Chinese scholars start to focus on the effect of CU traits on individuals’ social adjustment difficulties. For example, Fang and Wang found that CU traits positively predicted Chinese college students’ cyberbullying behavior [30]. Another study sampling Chinese primary school children showed that CU traits positively predicted children’s disciplinary problems [31]. Moreover, Zhang et al. found that CU traits not only positively predicted Chinese middle school students’ campus bullying behavior but also indirectly related to students’ campus bullying behavior through moral disengagement and guilt [32]. Exploring the link between CU traits and social adjustment in a Chinese cultural context may have particular cultural significance. Individuals with the same behavioral tendencies may have different experiences of social adjustment in different societies [33]. As reviewed above, compared with Western literature, research on CU traits in the Chinese context is relatively limited and the few existing Chinese studies have primarily sampled school-age children and adolescents [31,32]. From a psychopathological perspective, different cultures have different thresholds for determining problematic behaviors [34]. In contrast to the individualistic values espoused in the West, traditional Chinese culture emphasizes group harmony and interdependence as collectivistic values [35]. From this perspective, children with CU traits may face more severe social adjustment difficulties in China, as low empathy and indifferent behaviors may be more likely to be perceived as anti-collective and abnormal in the Chinese cultural context. In view of this, this study focused on 3–6 years old Chinese preschoolers and explored the relationship between CU traits and Chinese preschoolers’ social adjustment. Based upon prior research, it is hypothesized that CU traits would be positively associated with children’s negative social adjustment.

### 1.2. The Moderating Role of Teacher-Child Relationship

Despite the link between CU traits and social adjustment difficulties in many previous studies, children with high CU traits do not necessarily always exhibit social adjustment problems. There might be protective or risk factors that can buffer or exacerbate the social adjustment problems for children with CU traits. Developmental contextualism theory posits that children’s development is shaped through continual interactions with the surrounding environment [36]. Besides the family, kindergarten is one of the most exposed environments for children, and it is also an important place for the social development of young children. Teachers are an important part of children’s early school experiences and one of the main objects of attachment for young children [37]. Studies have shown that teacher-child relationships are significantly related to the internalizing and externalizing problems of young children [38,39,40]. Thus, the quality of the teacher-child relationship might moderate the link between CU traits and children’s social adjustment. The teacher-child relationship describes the perceptions and feelings of one party about their relationship with the other party [41]. The quality of the teacher-child relationship has two different dimensions: closeness and conflict [42]. Teacher-child closeness is characterized by positive interactions, open communication, and warm feelings between children and teachers. In contrast, teacher-child conflict is characteristic of negative interactions and negative affect between children and teachers [43]. According to the risk and protective factor framework by Masten, risk factors interact with protective factors to influence the development of individuals, in which protective factors buffer the negative effects of risk factors [44]. Also, much recent empirical work has evidenced the buffering role of a positive teacher-child relationship in the negative effect of risk factors on children. For example, Stoppelbein et al. found that teacher-child closeness can buffer the negative effect of adverse childhood experiences on CU traits of 6–14 years old children [45]. Jia et al. sampled 6–11 years old children and reported that a positive teacher-child relationship can reduce the social adjustment problems of children with conduct problems [46]. Research sampling preschool children also demonstrate that early teacher-child closeness can buffer the effects of children’s shyness on social adjustment difficulties [47]. These findings suggest that a close teacher-child relationship is a potential protective factor for children with CU traits. In addition, the differential susceptibility model postulates that individuals differ in their susceptibility to environmental influence, and children with heightened susceptibility are more sensitive to both negative and positive environmental conditions, especially in early childhood [48]. For example, Crum et al. focused on preschool children and found that teacher-child conflict exacerbated peer exclusion for children with CU traits [49]. Baroncelli et al. sampled 11–15 years old Italian children and found that teacher-child conflict enhanced insensitivity to teachers’ punishment of children with CU traits [50], which in turn engenders children’s higher conduct problems [51]. Consequently, the negative teacher-child relationship might be a risk factor for children with CU traits. As far as we know, there is a lack of research exploring how teacher-child relationships account for the association between CU traits and children’s social adjustment. Therefore, this study attempted to fill in this gap by examining whether the teacher-child relationship moderated the link between CU traits and preschool children’s social adjustment.

### 1.3. The Current Study

**Figure 1:**
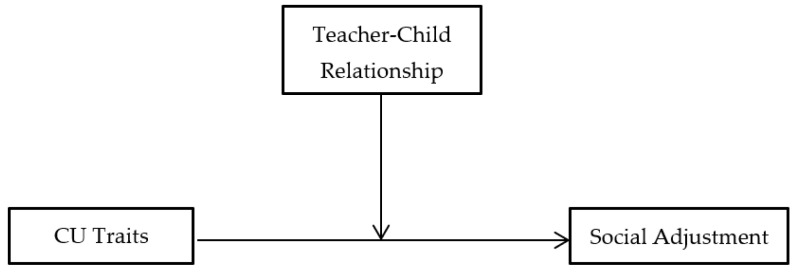
The hypothesized model.

As reviewed above, the present study aimed to explore the relationship between CU traits and Chinese preschoolers’ social adjustment as well as the moderating effect of the teacher-child relationship in this relation. The proposed model was presented in Figure 1. The specific hypotheses were as follows: (1) CU traits would be positively associated with Chinese preschool children’s social adjustment problems (i.e., aggressive behavior, prosocial behavior, asocial behavior, and peer exclusion); (2) Teacher-child relationship would moderate the relationship between CU traits and social adjustment. Teacher-child closeness would buffer the negative social adjustment of children with CU traits. Teacher-child conflict would exacerbate the negative social adjustment of children with CU traits.

## 2. Methods

### 2.1. Participants

Participants were 484 children aged 3–6 years old (227 boys, 46.9%, *M*age = 5.56, *SD* = 0.96) from two public kindergartens in Shanghai. Informed consent forms were collected from both teachers and parents, yielding a 98% return rate.

Nearly 6.2% of the mothers and 6.2% of the fathers had completed middle school; 9.5% of the mothers and 9.5% of the fathers had completed high school; 24.2% of the mothers and 16.3% of the fathers had completed junior college; 49.6% of the mothers and 52.9% of the fathers had earned a bachelor’s degree; and 6.6% of the mothers and 11.2% of the fathers had earned a postgraduate degree. Maternal and paternal scores were averaged to create a broader measure of parental education (with higher scores representing higher education).

### 2.2. Data Collection

The present study used a cross-sectional design. Mothers of the child participants reported their children’s CU traits and teachers reported their relationship with each individual child and rated children’s social adjustment based on daily observations.

### 2.3. Measures

#### 2.3.1. CU Traits

Children’s CU traits were measured with the Inventory of Callous-Unemotional Traits (ICU) [52]. The Chinese version of the ICU contains 19 items, and has been demonstrated to have a two-factor structure in the Chinese preschooler population and presented psychometric soundness [53]. The questionnaire includes the subscale of callousness (9 items, e.g., ‘Does not show emotions.’), and uncaring (10 items, e.g., ‘Does not care who he/she hurts to get what he/she wants.’). Callousness involves an indifferent attitude toward things and others, whereas uncaring involves not caring about the behavior and performance of oneself or others. The ICU was measured on a 4-point Likert scale ranging from definitely does not apply (coded as 0) to definitely applies (coded as 3). The average of all the items within the ICU was computed to represent children’s levels of CU traits, and 12 questions reverse scoring (e.g., ‘Expresses his/her feelings openly.’). The internal consistency coefficient for the ICU was 0.88.

#### 2.3.2. Social Adjustment

Children’s social adjustment was measured with the Child Behavior Scale (CBS) [16]. The Chinese version of the CBS has been validated with Chinese preschoolers and contains 35 items [54]. Of particular interest for the present study was the subscale of Aggressive behavior (7 items, e.g., ‘Fights’), Asocial behavior (6 items, e.g., ‘Prefers to play alone’), Prosocial behavior (7 items, e.g., ‘Kind toward peers’), and Peer exclusion (7 items, e.g., ‘Peers refuse to let child play’). Items measured on a 3-point Likert scale (1 = “definitely does not apply”, 3 = “definitely applies”). All items within each subscale were averaged to obtain a score for each dimension, with higher scores representing higher levels of specific behaviors. In the present study, Cronbach’s *α* values for the aggressive, asocial, prosocial, and peer exclusion scales were 0.90, 0.88, 0.90, and 0.93, respectively.

#### 2.3.3. Teacher-Child Relationship

The Student Teacher Relationship Scale (STRS) has been frequently used in teachers’ perceptions of their relationships with children [55]. The Chinese version of the STRS has shown adequate reliability and validity [56]. Of particular interest for the present study was the subscale of Conflict (12 items, e.g., ‘This child and I always seem to be struggling with each other’) and Closeness (11 items, e.g., ‘This child spontaneously shares information about himself/herself’). Closeness assesses the teacher’s feelings of affection for and sense of communication with the child, whereas conflict measures the teacher’s perceptions of negativity and conflict with the child. Responses were recorded on a 5-point Likert scale (1 = “definitely does not apply” and 5 = “definitely applies”). The average of all items within each subscale was computed to obtain a score for each dimension. Higher scores represent higher levels of teacher-child closeness or conflict. Alpha values for teachers’ reports were 0.92 and 0.82 for conflict and closeness, respectively.

### 2.4. Statistical Analyses

The data were analyzed statistically using SPSS 26.0 (IBM Corp., Armonk, NY, USA). Considering the non-normal distribution of several variables, Spearman correlation was used to test the bivariate relationship and the Mann-Whiney test was used to explore gender differences among study variables [57,58]. Then, PROCESS Macro (Version 3.5) (New York, NY, USA) was employed to examine a multivariable linear regression model, which was used to examine the main effects and interaction between CU traits and the teacher-child relationship on social adjustment [59]. The moderating role has been assumed as statistically significant when the lower limit (Boot LLCI) and upper limit (Boot ULCI) of bootstrap results in 95% confidence interval are either below or above zero [59]. Next, Johnson-Neyman technology was adopted to further probe the significant interaction. This technique is a point estimation that more directly reflects the prediction of the dependent variable by the independent variable when the moderating variable takes on different values [60].

## 3. Results

### 3.1. Descriptive Statistics and Correlation Analysis

A series of Mann-Whitney tests were conducted to examine gender differences in children’s CU traits, social adjustment, and teacher-child relationship. Results showed that boys scored higher than girls in CU traits (*p* < 0.05), teacher-child conflict (*p* < 0.001), aggressive behavior (*p* < 0.001), asocial behavior (*p* < 0.01), and peer exclusion (*p* < 0.001), and girls scored higher than boys in teacher-child closeness (*p* < 0.05) and prosocial behavior (*p* < 0.001). In addition, children’s age was negatively related to children’s asocial behavior and positively related to children’s prosocial behavior (see Table 1). Therefore, children’s gender and age were used as control variables in later analyses.

As shown in Table 1, correlational analyses showed that CU traits were positively associated with aggressive behavior and asocial behavior, and negatively associated with children’s prosocial behavior. Teacher-child closeness was negatively related to asocial behavior and peer exclusion, and positively related to children’s prosocial behavior, whereas teacher-child conflict was positively related to aggressive behavior, asocial behavior, and peer exclusion, and negatively related to children’s prosocial behavior.

### 3.2. Regression Analyses

**Figure 2:**
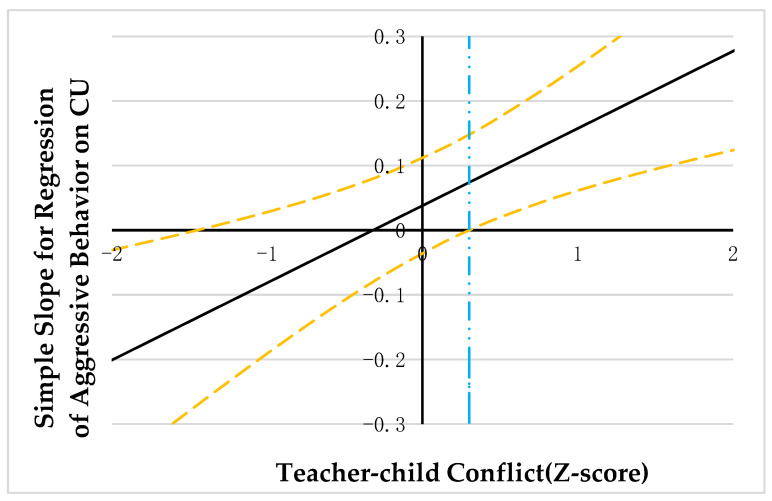
Johnson-Neyman regions of significance and confidence bands for mother-rated CU traits along teacher-child conflict in relation to aggressive behavior. Note. Solid diagonal line represents the regression coefficient for CU along with teacher-child conflict. Dashed diagonal yellow lines are confidence bands—upper and lower bounds of 95% confidence interval for CU coefficient along teacher-child conflict. The dashed vertical blue line indicates the point along teacher-child conflict at which the CU regression coefficient transitions from nonsignificance (left of the dashed vertical line) to statistical significance (right of dashed vertical line). The value of the dashed vertical line is 0.30.

**Figure 3:**
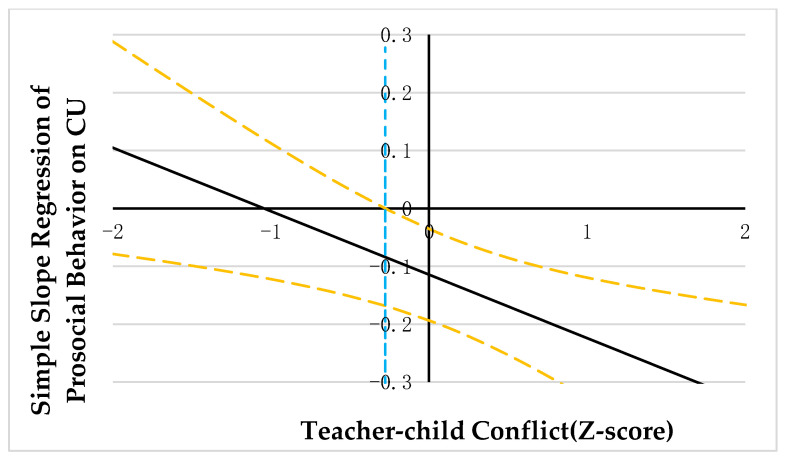
Johnson-Neyman regions of significance and confidence bands for mother-rated CU traits along teacher-child conflict in relation to prosocial behavior. Note. Solid diagonal line represents the regression coefficient for CU along with teacher-child conflict. Dashed diagonal yellow lines are confidence bands—upper and lower bounds of 95% confidence interval for CU coefficient along teacher-child conflict. The dashed vertical blue line indicates the point along teacher-child conflict at which the CU regression coefficient transitions from nonsignificance (left of dashed vertical line) to statistical significance (right of dashed vertical line). The value of the dashed vertical line is −0.28.

**Figure 4:**
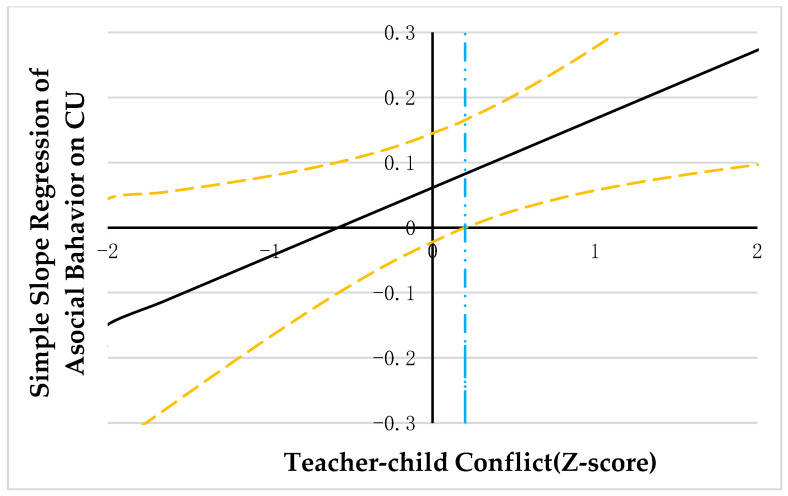
Johnson-Neyman regions of significance and confidence bands for mother-rated CU traits along teacher-child conflict in relation to asocial behavior. Note. Solid diagonal line represents the regression coefficient for CU along with teacher-child conflict. Dashed diagonal yellow lines are confidence bands—upper and lower bounds of 95% confidence interval for CU coefficient along teacher-child conflict. The dashed vertical blue line indicates the point along teacher-child conflict at which the CU regression coefficient transitions from nonsignificance (left of dashed vertical line) to statistical significance (right of dashed vertical line). The value of the dashed vertical line is 0.20.

PROCESS macro model 1 was employed to explore the moderating effect of teacher-child relationships [59]. As correlation analysis suggested that CU traits were significantly correlated with teacher-child conflict, the variables were diagnosed for collinearity issues in the regression analysis. The results showed that the tolerance for covariance was all greater than 0.20 and the VIF was all less than 5, suggesting that there was no covariance between CU traits and teacher-child conflict [61]. The results are summarized in Table 2 and Table 3. Johnson-Neyman technology was adopted to probe the significant interaction. Results were illustrated in Figure 2, Figure 3 and Figure 4.

According to Table 2, for the prediction of aggressive behavior, a significant main effect of gender was found, with girls scoring lower on aggressive behavior than boys. In addition, a significant main effect of teacher-child conflict (positive association) was found. The main effect for teacher-child conflict in the model for aggression behavior represents the association between teacher-child conflict and aggressive behavior in those with a score of zero on CU traits (e.g., those with average levels of CU traits, given that exposures were standardized). There were no significant main effects for age or CU traits. However, these effects were superseded by a significant two-way interaction effect (CU × Teacher-child conflict).

For the prediction of prosocial behavior, a significant main effect of gender was found, with girls scoring higher on prosocial behavior than boys. In addition, significant main effects of age (positive association), CU traits (negative association), and teacher-child conflict (negative association) were found. The main effect for CU traits in the model for prosocial behavior represents the association between CU traits and prosocial behavior in those with a score of zero on teacher-child conflict. Also, the main effect of teacher-child conflict in this model represents the association between teacher-child conflict and prosocial behavior in those with a score of zero on CU traits. These main effects were superseded by a negative significant two-way interaction effect (CU × Teacher-child conflict).

For the prediction of asocial behavior, a significant main effect of teacher-child conflict (positive association) was found. The main effect of teacher-child conflict in the model for asocial behavior represents the association between teacher-child conflict and asocial behavior in those with a score of zero on CU traits. However, there was no significant main effect of age, gender, and CU traits. The main effect was superseded by a significant two-way interaction effect (CU × Teacher-child conflict).

As reported in Table 3, the interaction effect between CU traits and teacher-child closeness on children’s aggressive behavior, prosocial behavior, and asocial behavior was not significant.

Results of the follow-up analysis (see Figure 2, Figure 3 and Figure 4) indicated that when the teacher-child conflict was >0.30, 0.20, and −0.28 units, separately, CU traits were positively associated with aggressive behavior and asocial behavior, and negatively associated with prosocial behavior. However, when the teacher-child conflict was ≤0.30, 0.20, −0.28 units, separately, CU traits were no longer significantly associated with aggressive behavior, asocial behavior, and prosocial behavior.

## 4. Discussion

This study examined the relationship between CU traits and social adjustment among 3–6 years old Chinese preschoolers as well as the moderating role of the teacher-child relationship in the association.

### 4.1. CU Traits and Children’s Social Adjustment

Consistent with previous studies, CU traits were associated with children’s conduct problems [20,22,24]. Results from the present study indicated that CU traits were positively associated with preschool children’s aggressive behavior and asocial behavior and negatively associated with children’s prosocial behavior. This result might be accounted for by several reasons. At first, Northam et al. sampled 2–8 years old children and found that children with a high level of CU traits display deficits in many aspects of emotional responses [25]. Emotional responses promote the development of prosocial behavior and empathy, and help children identify and perceive negative emotions in their peers [29]. Consequently, young children with a high level of CU traits have difficulty appreciating the emotions of others and tend to have a low level of prosociality. Second, a meta-analysis by Waller et al. showed that CU traits were moderately and highly negatively related to children’s guilt [23]. Yet, guilt can prompt young children to display more cooperative and prosocial behaviors [28]. Thus, children with high level of CU traits may exhibit more aggressive and less prosocial behaviors. In addition, Waller et al. found that 3–5 years old children’s fearless traits significantly and positively predicted children’s CU traits [23]. However, a fearless temperament can hinder children’s affective empathy and prosocial behavior [27]. Thus, fearless temperament probably makes children with a high level of CU traits violate disciplines without guilt. Also, Domínguez-Álvarez et al. conducted a study with Spanish preschool children and found that CU traits were significantly and negatively correlated with affiliative-type behaviors (e.g., deliberate eye gaze with others and interest in involving other children in play) [4]. This may explain the fact that children with high CU traits are less likely to initiate requests for socialization with peers and prefer to play alone instead. To sum up, children with high level of CU traits are more likely to exhibit higher levels of aggressive behavior and asocial behavior, and lower levels of prosocial behavior.

Inconsistent with the hypothesis, we did not find a significant association between CU traits and peer exclusion. Previous studies on CU traits and peer rejection presented mixed results. Some research demonstrated a significant association between the two variables [7,62], and some others did not find an association [20,63]. This may be due to subjective differences between different informants. For example, research that used peer reports had inconsistent results with research that used children’s self-reports [62,63]. This study used a teacher-reported approach, yet social interactions between children often occur outside of the adult’s view [64], and thus may have some influence on the result of this study. Furthermore, from a Contextual Developmental Perspective [65], children are more inclined to interact with children who have similar behavioral characteristics to themselves [66]. Research has shown that adolescents with CU traits are more inclined to associate with deviant and antisocial peers [9], and these adolescents have as many friends as regular adolescents [67]. Nevertheless, more research may be needed in the future to demonstrate the applicability of this result to early childhood.

### 4.2. The Moderating Role of Teacher-Child Relationship

Consistent with prior studies, the teacher-child relationship was closely related to children’s development [38,39,40]. The present study found that teacher-child closeness was negatively related to peer exclusion and asocial behavior, and positively related to children’s prosocial behavior. It is probable that teacher-child closeness can enhance children’s self-esteem, self-confidence, and social competence, with which children were more willing to actively participate in activities organized by schools and thereby they are more popular among peers [33]. In addition, this result supports the hypotheses derived from the Attachment Theory that a warm teacher-child relationship creates an emotional connection and helps children establish relationships with others and explore new environments [68]. Meanwhile, this study found that teacher-child conflict was negatively related to prosocial behavior and positively related to aggressive behavior, asocial behavior, and peer exclusion. This might be because teacher-child conflict might lead to children’s negative emotions. As a result, children might be unsatisfied with schools and are more likely to exhibit aggressive behavior, and asocial behavior and experience higher peer exclusion. In addition, this study found that CU traits were positively associated with teacher-child conflict and negatively associated with teacher-child closeness. This might be because children with CU traits have difficulty empathizing with others, lack emotions, and display higher aggressive and asocial behavior. They have more negative interactions with teachers resulting in more teacher-child conflict.

As hypothesized, teacher-child conflict moderated the relationship between CU traits and children’s aggressive behavior, asocial behavior, and prosocial behavior. Specifically, teacher-child conflict enhanced the aggressive and asocial behavior of children with CU traits and reduced their prosocial behavior. This result confirms a prior study by Crum et al., who conducted a 7-month longitudinal study with 1554 primary school students and found that high teacher-child conflict predicted greater adjustment difficulties for children with CU traits [49]. This finding supports the differential susceptibility model that a disadvantaged environment can lead to more negative adjustment for children with high susceptibility [48]. Undesirable interactions with teachers and negative affect pose a disadvantaged environment for children with CU traits, who tend to exhibit more conduct problems and less prosocial behaviors. Also, this result supports the double risk model that children at risk are more likely to display conduct problems in face of a disadvantaged environment [69]. When teachers are in tense conflict with children, they would adopt more coercive strategies and punishment toward children, which in turn exacerbates the aggressive and asocial behavior of children with CU traits. Meanwhile, teachers’ negative attitudes toward children can influence children’s peer acceptance [70]. As a result of the negative teacher-child interactions, teachers tend to provide negative feedback to children with CU traits. This might affect other children’s attitudes toward children with CU traits, resulting in lower acceptability among peers. Thus, children with CU traits are more likely to play alone and stay away from peers. In addition, the teacher-child relationship is relatively stable [71]. Frequent teacher-child conflicts lead to more negative assessments of children’s conduct problems [72]. Even if children with CU traits display appropriate behavior, long-term negative interactions with children can make teachers ignore these behaviors and withdraw positive feedback, all of which exacerbate the social adjustment difficulties of children with CU traits. As a result, CU traits and teacher-child conflict are two potential risk factors for children’s social adjustment.

However, contrary to our hypothesis, the moderating effect of teacher-child closeness on CU traits and children’s social adjustment was not identified in the present study. Although teacher-child closeness has a protective role for children at risk [73], children with CU traits tend to have a poor teacher-child relationship. Children with CU traits tend to show low levels of prosocial behavior, low empathy, and more conduct problems, making it difficult for them to establish warm and close relationships with teachers [74]. Children with CU traits may frequently experience negative interactions by displaying asocial behaviors, which may increase the likelihood of conflict and confrontational behavior with teachers later on [49]. Another possible explanation could be that close relationships with teachers can upgrade children’s confidence, which in turn leads children to challenge teachers’ authority. Children with CU traits have difficulty understanding others’ emotions, show limited affective empathy, and possess fearless temperament traits, making it hard for those children to share another person’s negative emotions. Also, they will probably not feel guilty or shameful for other persons’ pains. This might lead children with CU traits to believe that their conduct problems (e.g., aggressive behaviors) will not harm teacher-child relationships permanently and still display aggressive behavior and disciplinary problems [49]. Therefore, teacher-child closeness failed to buffer the negative adjustment of children with CU traits.

### 4.3. Limitations and Future Directions

Several limitations need to be acknowledged. At first, this study collected samples from one public kindergarten in Shanghai, China, which limited the generalizability to other regions. Future research can collect samples from different regions including both urban and rural areas in China. Also, this study was conducted in the context of Chinese culture, limiting the generalizability to other cultures, and future research could consider comparative studies of Chinese and Western cultures. Second, considering that preschool children are unable to complete written questionnaires, this study used parent and teacher-reported surveys measuring CU traits, children’s social adjustment, and teacher-child relationships. Thus, the data may be influenced by subjective factors of the subjects, resulting in social desirability bias. Future research can use multiple methods such as natural observations and peer evaluation with age-appropriate rating items. Third, this study used cross-sectional designs and could not reveal the causality and the directions of the relationship between CU traits and children’s social adjustment. Future research with longitudinal designs can further this line of research. Besides, there may be residual confounding given that gender and age were the only confounders included, and future studies could consider more residual confounding factors to make the research more scientifically rigorous. At last, given the small sample size, there may have been limited power to detect interactions that were smaller in magnitude, and future studies could expand the sample size to address this limitation.

## 5. Conclusions

In summary, the current study revealed that CU traits significantly predicted aggressive behavior, asocial behavior, peer exclusion, and prosocial behavior among young Chinese children. Additionally, teacher-child conflict moderated the relationship between CU traits and children’s social adjustment. Specifically, teacher-child conflict exacerbated the negative social adjustment of children with CU traits but no moderating effect of teacher-child closeness was identified for CU traits and children’s social adjustment. The present study constitutes a significant contribution to our understanding of the relations between CU traits and adjustment outcomes and the role of the teacher-child relationship in today’s Chinese children. As well, our findings may have practical implications for an early intervention program for Chinese children with CU traits. For example, teachers need to remember that the teacher-child relationship plays a very important role in the development of social adjustment. Specifically, teachers should continuously improve their emotion management skills to avoid conflicts with children with CU traits, especially in Chinese culture. Besides, considering that this study is the first to examine the relationship between early childhood CU traits and social adjustment in a Chinese cultural context, it may provide some empirical evidence for future cross-cultural research on CU traits in early childhood.

## Figures and Tables

**Table 1:** Means, standard deviations, and correlation coefficients for the study variables.

Variables	1	2	3	4	5	6	7	8	9	10
1. Chid age	-									
2. Child gender	0.02	-								
3. Parental education	0.03	0.04	-							
4. CU	−0.04	−0.11 *	−0.04	-						
5. Teacher-child closeness	−0.08	0.12 **	−0.05	−0.08	-					
6. Teacher-child conflict	0.01	−0.20 ***	0.02	0.17 ***	−0.19 ***	-				
7. Aggressive behavior	0.05	−0.26 ***	−0.04	0.10 *	0.03	0.47 ***	-			
8. Prosocial behavior	0.13 **	0.20 ***	−0.04	−0.15 **	0.55 ***	−0.46 ***	−0.20 ***	-		
9. Asocial behavior	−0.13 **	−0.13 **	0.04	0.09 *	−0.27 ***	0.31 ***	0.26 ***	−0.38 ***	-	
10. Peer exclusion	−0.08	−0.26 ***	−0.01	0.08	−0.26 ***	0.38 ***	0.42 ***	−0.35 ***	0.55 ***	-
*M*	4.88	-	-	0.66	4.04	1.59	1.10	2.38	1.12	1.10
*SD*	0.88	-	-	0.44	0.68	0.87	0.28	0.60	0.30	0.31
Skewness	−0.12	-	-	0.68	−1.15	1.70	3.33	−0.74	3.10	3.10
Kurtosis	−1.05	-	-	0.19	1.63	4.64	11.55	0.11	10.37	9.57

Note. *** *p* < 0.001; ** *p* < 0.01; * *p* < 0.05. Gender (boys = 1, girls = 2).

**Table 2:** Effects of CU traits, teacher-child conflict in relation to indices of social adjustment.

Social Adjustment Variables				
Predictor	B	SE	t	95% CI
**Aggressive behavior**				
Child age	0.03	0.04	0.64	[−0.05, 0.11]
Child gender	−0.38 ***	0.08	−5.01	[−0.53, −0.23]
CU	0.04	0.04	0.99	[−0.04, 0.11]
T-C conflict	0.52 ***	0.04	13.58	[0.44, 0.59]
CU × T-C conflict	0.12 **	**0.04**	**3.27**	**[0.05, 0.19]**
**Prosocial behavior**				
Child age	0.09 *	0.04	2.06	[0.00, 0.18]
Child gender	0.37 ***	0.08	4.55	[0.21, 0.52]
CU	−0.11 **	0.04	−2.85	[−0.19, −0.04]
T-C conflict	−0.35 ***	0.04	−8.69	[−0.43, −0.27]
CU × T-C conflict	−0.11 **	**0.04**	**−2.75**	**[−0.19, −0.03]**
**Asocial behavior**				
Child age	−0.05	0.05	−1.09	[−0.14, 0.04]
Child gender	−0.16	0.08	−1.86	[−0.32, 0.01]
CU	0.06	0.04	1.46	[−0.02, 0.14]
T-C conflict	0.33 ***	0.04	7.77	[0.25, 0.42]
CU × T-C conlfict	0.11 *	**0.04**	**2.52**	**[0.02, 0.19]**
**Peer exclusion**				
Child age	−0.01	0.04	−0.31	[−0.10, 0.07]
Child gender	−0.33 ***	0.08	−4.17	[−0.49, −0.18]
CU	0.00	0.04	−0.12	[−0.08, 0.07]
T-C conflict	0.43 ***	0.04	10.64	[0.35, 0.51]
CU × T-C conlfict	0.05	0.04	1.25	[−0.03, 0.13]

Note. All continuous variables were standardized before entering the regression. *** p < 0.001; ** p < 0.01; * p < 0.05. All significant interaction effects are presented in bold.

**Table 3:** Effects of CU traits, teacher-child closeness in relation to indices of social adjustment.

Social Adjustment Variables				
Predictor	B	SE	t	95% CI
**Aggressive behavior**				
Child age	0.04	−0.05	0.79	[−0.56, 0.14]
Child gender	−0.52 ***	0.09	−5.89	[−0.70, −0.35]
CU	0.10 *	0.04	2.16	[0.01, 0.18]
T-C closeness	−0.10 *	0.05	−2.16	[−0.19, −0.01]
CU × T-C closeness	0.01	0.04	0.28	[−0.07, 0.09]
**Prosocial behavior**				
Child age	0.05	0.04	1.16	[−0.03, 0.13]
Child gender	0.35 ***	0.07	4.90	[0.21, 0.50]
CU	−0.11 **	0.04	−2.97	[−0.18, −0.04]
T-C closeness	0.54 ***	0.04	14.42	[0.46, 0.61]
CU × T-C closeness	−0.05	0.03	−1.46	[−0.12, 0.02]
**Asocial behavior**				
Child age	−0.01	0.05	−0.28	[−0.10, 0.08]
Child gender	−0.16 *	0.08	−1.99	[−0.32, 0.00]
CU	0.07	0.04	1.64	[−0.01, 0.15]
T-C closeness	−0.41 ***	0.04	−9.73	[−0.49, −0.33]
CU × T-C closeness	0.06	0.04	1.67	[−0.01, 0.14]
**Peer exclusion**				
Child age	0.01	0.05	0.29	[−0.08, 0.11]
Child gender	−0.40 ***	0.08	−4.73	[−0.57, −0.23]
CU	0.01	0.04	0.34	[−0.07, 0.10]
T-C closeness	−0.29 ***	0.04	−6.75	[−0.38, −0.21]
CU × T-C closeness	0.04	0.04	0.95	[−0.04, 0.12]

Note. All continuous variables were standardized before entering the regression. *** p < 0.001; ** p < 0.01; * p < 0.05.

## Data Availability

The data presented in this study are available on request from the corresponding author.
